# Death of Professor Cooper

**Published:** 1862-12

**Authors:** 


Death of Professor Cooper.—Our readers will hear with regret of the death of Dr. E. S. Cooper, late editor of the *Medical Press*, and Professor of Anatomy and Surgery in the University of the Pacific. Dr. Cooper was a bold operator, devoted to his profession, and of untiring energy. The medical faculty, of which he was a prominent member, has, by his untimely demise, sustained a severe loss. He died on the 13th ult., at 20 minutes before 9 A. M., in the fortieth year of his age. His funeral was attended by a large concourse of mourning friends.—*Pacific Medical and Surgical Journal.*

